# The Treatment Outcome of Smart Device–Based Tinnitus Retraining Therapy: Prospective Cohort Study

**DOI:** 10.2196/38986

**Published:** 2023-01-12

**Authors:** Myung-Whan Suh, Moo Kyun Park, Yoonjoong Kim, Young Ho Kim

**Affiliations:** 1 Department of Otorhinolaryngology Seoul National University Hospital Seoul Republic of Korea; 2 Sensory Organ Research Institute Seoul National University Medical Research Center Seoul Republic of Korea; 3 Department of Otorhinolaryngology-Head and Neck Surgery Seoul Metropolitan Government-Seoul National University Boramae Medical Center Seoul Republic of Korea; 4 Department of Otorhinolaryngology-Head and Neck Surgery Chungbuk National University Hospital Cheongju-si Republic of Korea

**Keywords:** tinnitus, tinnitus retraining therapy, smart device, sound therapy, rehabilitation, therapy, tablet application, app-based, therapy, digital therapy, device-based therapy

## Abstract

**Background:**

Tinnitus retraining therapy (TRT) is a standard treatment for tinnitus that consists of directive counseling and sound therapy. However, it is based on face-to-face education and a time-consuming protocol. Smart device–based TRT (smart-TRT) seems to have many advantages, but the efficacy of this new treatment has been questioned.

**Objective:**

The aim of this study was to compare the efficacy between smart-TRT and conventional TRT (conv-TRT).

**Methods:**

We recruited 84 patients with tinnitus. Results were compared between 42 patients who received smart-TRT and 42 control participants who received conv-TRT. An interactive smart pad application was used for directive counseling in the smart-TRT group. The smart pad application included detailed education on ear anatomy, the neurophysiological model of tinnitus, concept of habituation, and sound therapy. The smart-TRT was bidirectional: There were 17 multiple choice questions between each lesson as an interim check. The conv-TRT group underwent traditional person-to-person counseling. The primary outcome measure was the Tinnitus Handicap Inventory (THI), and the secondary outcome measure was assessed using a visual analogue scale (VAS).

**Results:**

Both treatments had a significant treatment effect, which comparably improved during the first 2 months. The best improvements in THI were –23.3 (95% CI –33.1 to –13.4) points at 3 months and –16.8 (95% CI –30.8 to –2.8) points at 2 months in the smart-TRT group and conv-TRT group, respectively. The improvements on the VAS were also comparable: smart-TRT group: –1.2 to –3.3; conv-TRT: –0.7 to –1.7.

**Conclusions:**

TRT based on smart devices can be an effective alternative for tinnitus patients. Considering the amount of time needed for person-to-person counseling, smart-TRT can be a cost-effective solution with similar treatment outcomes as conv-TRT.

## Introduction

Tinnitus retraining therapy (TRT) is a habituation therapy that can alleviate tinnitus-induced distress by means of directive counseling and sound therapy [1,2]. Despite the various attempts to cure tinnitus, currently there is no treatment that can completely eliminate tinnitus. Psychological and behavioral interventions such as cognitive behavioral therapy and TRT have been applied as alternatives. According to the 2014 guideline from the American Academy of Otolaryngology–Head and Neck Surgery, clinicians should “educate” patients with persistent, bothersome tinnitus about management strategies [3]. Also, clinicians may recommend “sound therapy” to patients with persistent, bothersome tinnitus [3]. These 2 strategies constitute the basis of TRT. Although high-quality randomized clinical trials are still lacking, it has been proposed in a Cochrane Database systematic review that TRT is more effective than sound masking [4].

One problem with TRT is that it is very time-consuming. With classic TRT, patients undergo at least 3 or 4 sessions of extensive, one-on-one, 60-minute, directive counseling that includes education about the auditory system, brain function, and Jastreboff’s neurophysiological model [2,5]. Due to the long and extensive counseling, it takes a lot of manpower and time. This problem is also associated with the high cost of TRT. In order to overcome this problem, group counseling [6] and simplified TRT [7] have been proposed by several researchers. Simplified TRT is efficient in that the counseling is short (30 minutes) and it omits the lengthy explanation about the anatomy and physiology of hearing. According to previous results, it seems that the treatment effect of simplified TRT is similar to that of classic TRT [7].

To further save manpower and time, we have come up with a TRT using interactive applications and smart devices (smart pad and smartphone). Instead of the classic one-on-one, 60-minute counseling, patients may engage with an application that delivers all the directive counseling content. A tablet computer or smart pad such as an iPad can be used for the bidirectional and intuitive operation. Traditional audiovisual devices such as a television are unidirectional: They can provide information to the user but cannot give any feedback. With the help of recent technology, we can now build applications that are bidirectional: The system can ask questions and assess progress, mimicking one-on-one counseling [8]. This system may also be beneficial for patients, since the cost of TRT can be reduced and there are fewer constraints around time and space [9,10]. Sound therapy can also be provided using an application or mp3 file installed on the patient’s smartphone.

Smart device–based TRT (smart-TRT) seems to have many advantages, but the efficacy of this new treatment has been questioned. Especially, the low level of human contact during TRT may lead to insufficient engagement with the educational intervention [11]. If this new technology is to be recommended to patients, the treatment outcome should be similar or better than conventional TRT (conv-TRT). The aim of this study was to compare the efficacy between smart-TRT and conv-TRT.

## Methods

### Participants

**Figure 1:**
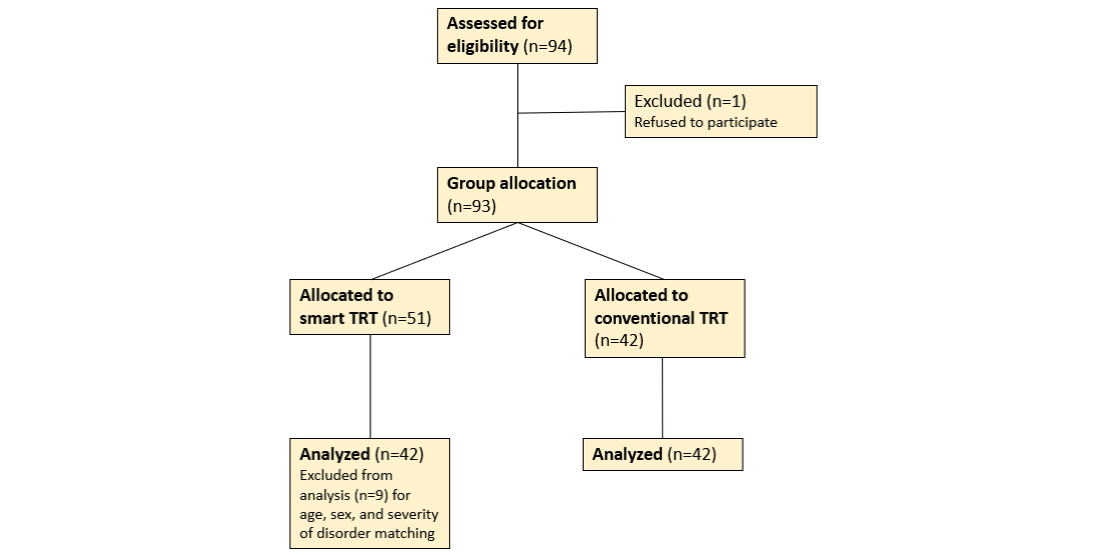
Flow diagram of the study design. TRT: tinnitus retraining therapy.

This study was a prospective trial with 2 arms. Both groups were recruited from the same institute during the same period, but group allocation was not randomized. The participants freely selected between the newly developed intervention or the conventional treatment. Of the 94 participants assessed for eligibility, 1 participant was excluded because the person refused to participate. Of the remaining 93 participants, 51 participants were allocated to the smart-TRT group, and 42 participants were allocated to the conv-TRT group (Figure 1). All participants underwent the intervention, and no one dropped out. We excluded 9 participants from the final analysis in order to match the 2 groups in terms of age, sex, and severity of tinnitus. The inclusion criteria were patients who were older than 20 years and had experienced chronic essential tinnitus for more than 6 months. Patients with vascular tinnitus, posttraumatic tinnitus, psychological disorders, severe hearing loss, sleep disorders, dementia, organic brain disorders, substance abuse, chronic renal failure, or uncontrolled malignancy were excluded from the study. Normal middle ear status was confirmed via audiogram and otoscopy, and we screened for abnormal psychological conditions such as depression, anxiety, and insomnia using the validated version of the Beck Depression Inventory (BDI) for depression [12,13], the State-Trait Anxiety Inventory (STAI) for anxiety [14], and the Pittsburgh Sleep Quality Index (PSQI) for sleep quality [15].

### Ethics Approval

This study was approved by the Seoul National University Hospital Institutional Review Board (IRB no. 1207-112-419) and was conducted according to the tenets of the Declaration of Helsinki. All participants provided written informed consent.

### Directive Education

For the smart-TRT group, 3 different interactive smart pad applications were prepared for the 3 directive education sessions. Each application was a composite of numerous video clips. Two screens were displayed on the smart pad: a big screen that showed illustrations or cartoons and a small screen that showed the face of the speaker (1st session, MWS; 2nd session, MKP; 3rd session, YHK). The 3 smart pad applications were presented to the patients with a 1-month interval between each at the clinic. We did not allow the participants to use these applications at home by themselves, to allow a fair comparison with the control group.

The smart pad applications included detailed education on the anatomy and physiology of the ear and auditory pathways, the perception of sound in the auditory cortex, “selective” listening, why tinnitus becomes a problem, the misconception that tinnitus causing hearing difficulties, an explanation of habituation as a goal, subconscious processing of auditory stimuli, “filtering” and “blocking” auditory stimuli from reaching consciousness, how to apply “sound therapy,” the neurophysiological model, and homework for the patient. Although the first session explained every aspect of these points, the second and third sessions reviewed the first session and added some new points with further examples. The smart-TRT was bidirectional: There were 17 multiple choice questions between each lesson for an interim check. The questions were mandatory, and the education session did not proceed if the patient did not respond. After the patient’s response, the correct answer was provided with further explanation why the answer was correct or incorrect. Since the patient’s response to each question was quite variable, the duration of the directive education differed between participants. It took at least 45 minutes for a patient to complete the first education session if the patient answered all the questions correctly and quickly. It took at least 25 minutes for a patient to complete the second and third education sessions. For some patients who had difficulty understanding the directive education, it could take more than 1 hour to complete 1 session.

For the conv-TRT group, simplified group (1-4 patients/session) counseling was provided by a single clinician (MWS). The counselor in the conv-TRT group was identical to the first speaker in the smart-TRT group (MWS). The contents and teaching materials for the directive counseling were also identical between the 2 groups. It took about 45 minutes to 60 minutes for a patient to complete the first session. It took about 10 minutes to 20 minutes for a patient to complete the second and third education sessions. The second and third sessions were rather short in the conv-TRT group, because most of the essential information and strategies were already well-covered. It took less time to review the last session and add new knowledge and encourage higher levels of motivation. Other than these points, all the other treatment and follow-up conditions were the same in the 2 groups.

### Sound Therapy

The same sound source file (white noise) was provided to the patients in both groups. The patients used their own smartphone or a portable mp3 player to play the sound. If the patient was familiar with using smartphone applications, a sound therapy application that had been built by our group was installed on their smartphone. We instructed the patients in both groups to use the sound therapy device at the level of the mixing point for at least 6 hours a day.

### Outcomes and Statistical Analysis

To calculate the sample size, the study was powered at 80% with a type I error of 5%. We assumed that a 5.9 difference in the Tinnitus Handicap Inventory (THI) score with an SD of 8 between the treatment groups was significant based on the study by Kaldo et al [16]. Assuming a loss of 30%, the number of patients needed for each group was 42 (84 patients total).

The primary outcome measure was the THI score. The change in the THI score was defined as ΔTHI = postTHI score – preTHI score. The secondary outcome measure was a visual analogue scale (VAS) score to quantify awareness of tinnitus, loudness of tinnitus, annoyance caused by tinnitus, and the effects of tinnitus on daily life [17-20]. The change in the VAS score was defined as ΔVAS = postVAS score – preVAS score. The effects of TRT were assessed based on changes in the THI and VAS scores at 0, 1, 2, and 3 months after the TRT. Continuous variables are expressed as mean (SD), and all statistical analyses were performed using SPSS version 16.0 (SPSS Inc, Chicago, IL). A repeated measure analysis of variance was used to evaluate the effect of time, group, and interaction between time and group. We used *t* tests to compare continuous variables and chi-square tests to compare categorical variables. *P* values <.05 were considered to indicate statistical significance.

## Results

### Demographics

Among the 84 participants (mean age 57.9, SD 11.1 years; 40 men and 44 women), the mean baseline THI was 48.7 (SD 20.9). The baseline clinical characteristics of the smart-TRT group and conv-TRT group are summarized in Table 1. There were no differences in age, gender, affected ear, baseline THI, baseline VAS, baseline STAI, baseline BDI, baseline PSQI, loss to follow-up rate, and pure tone audiometry threshold.

**Table 1:** Baseline characteristics.

Characteristics			Smart TRT^a^ (n=42)		Conventional TRT (n=42)		*P* value	
Age (years), mean (SD)			55.8 (12.0)		59.9 (9.9)		.09	
**Gender, n (%)**								.38
	Male	22 (52)		18 (43)				
	Female	20 (48)		24 (57)				
**Side, n (%)**								.72
	Right	9 (21)		10 (24)				
	Left	12 (29)		16 (38)				
	Both	15 (36)		12 (29)				
	Head or unclear	6 (14)		4 (10)				
Baseline Tinnitus Handicap Inventory, mean (SD)			46.9 (20.7)		50.5 (21.1)		.43	
**Baseline VAS^b^ (awareness), mean (SD)**								
	Awareness	7.1 (3.4)		7.2 (2.9)		.92		
	Annoyance	5.9 (2.7)		6.6 (2.8)		.22		
	Loudness	5.8 (2.4)		6.8 (2.3)		.06		
	Effect on daily life	4.4 (2.3)		4.9 (2.9)		.41		
**State-Trait Anxiety Inventory, mean (SD)**								
	X1	44.1 (9.1)		46.0 (10.9)		.39		
	X2	43.0 (7.2)		45.7 (12.0)		.24		
Beck Depression Inventory, mean (SD)			13.4 (9.6)		15.1 (10.0)		.43	
Pittsburgh Sleep Quality Index, mean (SD)			7.8 (3.9)		8.9 (5.8)		.35	
Loss to follow-up at 3 months, n (%)			19 (55)		19 (55)		.99	
**PTA^c^ threshold (dB HL^d^, mean (SD)**								
	Right	23.2 (14.6)		25.6 (16.8)		.49		
	Left	24.0 (15.4)		28.9 (20.6)		.22		

^a^TRT: tinnitus retraining therapy.

^b^VAS: visual analogue scale.

^c^PTA: pure-tone audiometry; mean 6-tone average = (500 Hz + 2*1000 Hz + 2*2000 Hz + 4000 Hz)/6.

^d^HL: hearing loss.

### Primary Outcome Measure: THI

**Figure 2:**
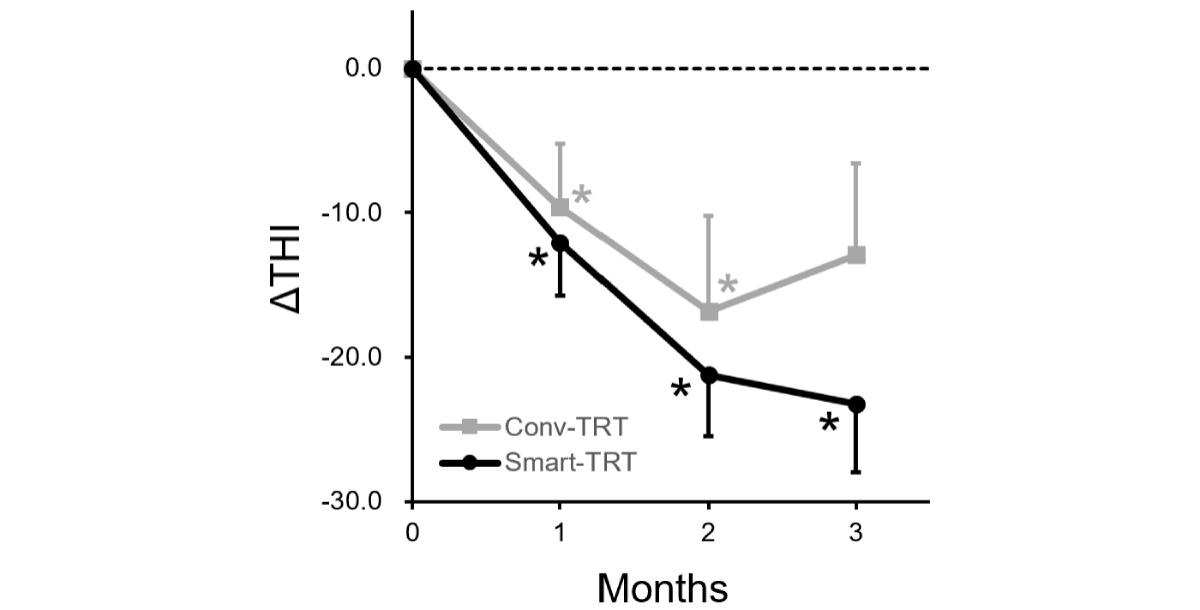
Mean (SD: error bars) change in the Tinnitus Handicap Inventory (THI) score (ΔTHI) as a function of time. Conv-TRT: conventional tinnitus retraining therapy; Smart-TRT: smart device tinnitus retraining therapy. *P<.05.

Figure 2 shows the mean ΔTHI as a function of time. The best ΔTHI score was –23.3 points (95% CI –33.1 to –13.4) at 3 months and –16.8 points (95% CI –30.8 to –2.8) at 2 months in the smart-TRT group and conv-TRT group, respectively. In both groups, the THI score significantly improved over time (within-participant effect: *F*_1.8,42.1_=10.741, *P*<.001), but there was no difference in the treatment outcome between the 2 groups (between-participant effect: *F*_1,24_=0.094, *P*=.76). Also, there was no interaction between time and group (time x group effect: *F*_1.8,42.1_=0.773, *P*=.45). That is, the pattern of gradual improvement as well as the outcome were similar between smart-TRT and conv-TRT.

When each time point was evaluated, a significant reduction in the THI score was found in the smart-TRT group at 1 month (t_27_=–3.312, *P*=.003), 2 months (t_23_=–5.040, *P*<.001), and 3 months (t_18_=–4.947, *P*<.001). A significant reduction was also found in the conv-TRT group at 1 month (t_30_=2.183, *P*=.04) and 2 months (t_16_=–2.549, *P*=.02). The treatment effect was marginal (t_18_=–2.037, *P*=.057) after 3 months of conv-TRT (Table 2).

**Table 2:** Treatment outcome at 3 months.

Outcomes		Smart TRT^a^ group			Conventional TRT group	
		Results, mean (95% CI)	*P* value^b^	Results, mean (95% CI)		*P* value^b^
Primary outcome measure: change in the Tinnitus Handicap Inventory		–23.3 (–33.1 to –13.4)	<.001	–12.9 (–26.3 to 0.41)		.057
**Secondary outcome measure: change in the visual analogue scale**						
	Awareness of tinnitus	–3.28 (–4.99 to –1.57)	.001	–1.37 (–2.86 to 0.12)		.07
	Annoyance due to tinnitus	–2.89 (–4.24 to –1.54)	<.001	–1.74 (–3.19 to –0.28)		.02
	Loudness of tinnitus	–1.22 (–2.41 to –0.03)	.045	–0.74 (–1.80 to 0.33)		.16
	Effect on daily life by tinnitus	–2.67 (–3.93 to –1.40)	<.001	–1.26 (–2.47 to –0.05)		.04

^a^TRT: tinnitus retraining therapy.

^b^1-sample *t* test compared with 0.

### Secondary Outcome Measure: VAS

**Figure 3:**
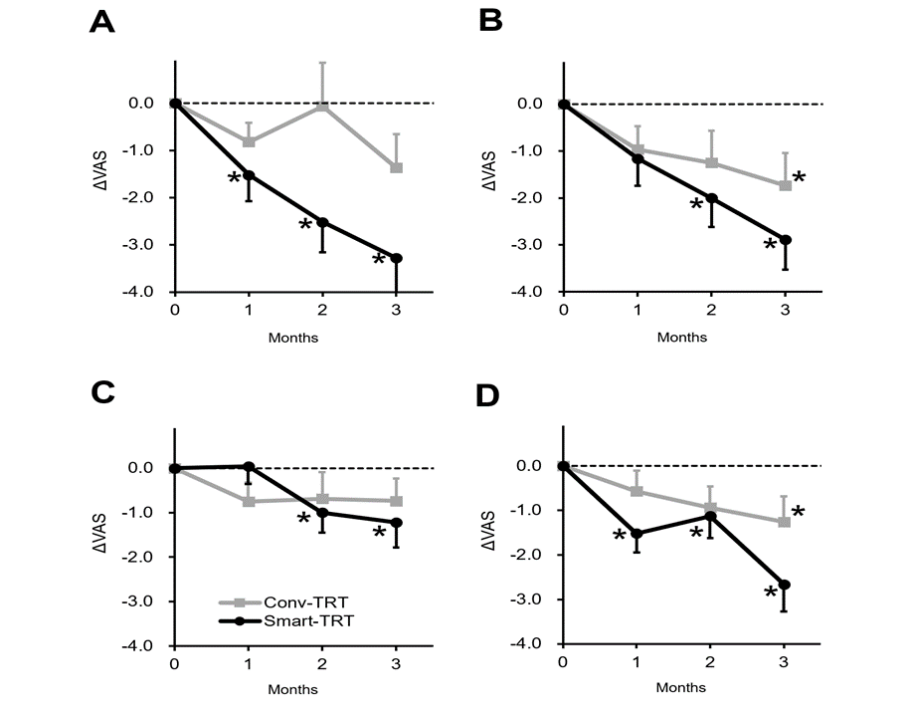
Mean (SD: error bars) change in the visual analogue scale (VAS) scores (ΔVAS) as a function of time: (A) awareness of tinnitus, (B) annoyance caused by tinnitus, (C) loudness of tinnitus, (D) effect of tinnitus on daily life. Conv-TRT: conventional tinnitus retraining therapy; Smart-TRT: smart device tinnitus retraining therapy. *P<.05.

Figure 3 shows the mean ΔVAS as a function of time. In both groups, the VAS significantly improved over time (within-participant effect) in 3 VAS categories: awareness of tinnitus (*F*_2.2,4.4_=6.667, *P*=.002), annoyance caused by tinnitus (*F*_3,66_=4.358, *P*=.007), and effect of tinnitus on daily life (*F*_3,66_=4.288, *P*=.008). There was no significant change in the loudness of tinnitus over time (*F*_2.4,51.9_=0.795, *P*=.48).

There were no differences in the treatment outcome between the 2 groups (between-participant effect) in all 4 VAS categories: awareness of tinnitus (*F*_1,22_=1.196, *P*=.29), annoyance caused by tinnitus (*F*_1,22_=2.507, *P*=.13), loudness of tinnitus (*F*_1,22_=0.163, *P*=.96), and effect of tinnitus on daily life (*F*_1,22_=2.518, *P*=.13). Also, there were no significant interactions between time and group (time x group effect): awareness of tinnitus (*F*_2.2,47.7_=0.790, *P*=.47), annoyance caused by tinnitus (*F*_3,66_=1.371, *P*=.26), loudness of tinnitus (*F*_2.4,51.9_=0.568, *P*=.60), and effect of tinnitus on daily life (*F*_3,66_=1.606, *P*=.20).

When the ΔVAS was evaluated within each time point, a significant treatment effect was found in the smart-TRT group within 1 month to 2 months. The maximum treatment effect was found at the last follow-up time point (3 months; Table 2). That is, ΔVAS for awareness of tinnitus (t_17_=–4.038, *P*=.001), annoyance caused by tinnitus (t_17_=–4.506, *P*=.045), loudness of tinnitus (t_17_=–2.170, *P*<.001), and effect of tinnitus on daily life (t_17_=–4.038, *P*=.001) were significantly different from 0 (1-sample *t* test) at 3 months. A similar pattern was found in the conv-TRT group, but the significant improvements were only found in ΔVAS for annoyance caused by tinnitus (t_10_=–2.511, *P*=.02) and effect of tinnitus on daily life (t_18_=–2.191, *P*=.04) after 3 months (Figure 3).

## Discussion

### Principal Findings

From this study, we were able to show that the treatment outcome of smart-TRT is similar to that of conv-TRT. That is, both treatments had a significant treatment effect that comparably improved over time. As for the primary outcome measure (ΔTHI), the improvement was –23.3 in the smart-TRT group and –16.8 in the conv-TRT group (significant improvement over time within both groups, but no difference between groups). These improvements are not only statistically significant but also clinically significant, given that a change in THI score of 7 or greater is clinically meaningful [21]. The secondary outcome (ΔVAS) was also similar between the 2 groups, with a slight advantage in the smart-TRT group (between –1.2 and –3.3) compared with the conv-TRT group (between –0.7 and –1.7). At 3 months, the THI score slightly deteriorated in the conv-TRT group, while the effect lasted in the smart-TRT group. Although the *P* value was marginal, the ΔTHI score at 3 months was not different from baseline. This finding may imply a deterioration of the treatment result after 3 months. Meanwhile, the secondary outcome measure (VAS scores for annoyance and effect on daily life) showed a steady decrease at 3 months in both groups. It seems that TRT delivered via smart devices can be an alternative treatment for tinnitus patients with similar treatment effects.

The attempt to use smart devices [22] and internet-based audiovisual media [8] for educational interventions for patients with chronic health conditions is a common trend. For example, video-based education and interactive games have been used for patients with type 2 diabetes mellites [23,24]. Multimedia-based animations and quizzes have helped patients with obesity [25]. Osteoarthritis has also been managed with educational modules consisting of text and video [26]. The use of information and communication technology (ICT) for health-related purposes was able to help disease management by facilitating access to health information and helping to increase understanding of the disease [8,27]. Directive counseling in TRT is more complicated than these examples because it is bidirectional. The counselor must understand the individual condition of each patient and tailor the instructions and counseling content depending on the patient’s response. However, recent studies postulated that ICT-based interaction can provide not only 1-way educational interventions but also 2-way, interactive counseling via electronic counseling or e-counseling [8]. We agree that smart-TRT can be limited in this bidirectional interactive communication. Also, the low level of human contact may decrease the efficacy and motivation [11]. However, there seems to be other advantages that can balance these limitations.

The biggest advantage of smart-TRT is that it is cost-effective. Since the directive counseling is performed by an application installed on a smart pad, the health provider does not have to spend much time with each patient. Delivering the main idea of TRT and checking whether the patient is making good progress can be done via the smart pad. During this study, the health provider only had to answer questions after each smart-TRT course. The high cost-effectiveness of ICT-based management has been proven for other interventions such as lifestyle modification [9], weight gain prevention [10], and smoking cessation [28]. There is no cost-effectiveness analysis for smart-TRT yet. However, we think it will at least reduce medical personnel expenses for each counseling session. Moreover, by using multiple smart pad devices, many patients can simultaneously undergo directive counseling under the supervision of 1 health provider. Another advantage of smart-TRT is that it can be used for contactless health care and telemedicine. W Beukes et al [29] reported that Internet-based cognitive behavioral therapy for tinnitus could overcome accessibility barriers. The COVID-19 outbreak has greatly changed our way of living as well as how we obtain medial information [30]. We think smart-TRT can be a contactless solution for tinnitus during such difficult times.

The smart pad interface and interactive nature of the multimedia content seem to be critical to the outcome of smart-TRT. The high efficacy of ICT-based education is now generally accepted [31]. As a result, ICT-based courses in college and university have increased by 440% during the past decade [32]. However, there are 2 differences between patients with tinnitus and higher education students. First, the input method and device interface can be a barrier to some patients with tinnitus. Most patients with tinnitus are old and not completely comfortable with a computer, mouse, trackpad, and keyboard. Smart devices using a touch screen interface may play an important role in such situations. That is, given the simplicity and self-explanatory nature, most patients can easily learn how to operate and interact with a smart pad. During our study, it took less than 2 minutes to explain how to use the device, despite some patients having no experience with smart pads. Second, the desire to adopt new information, attitudes, and ideas is much lower in patients with tinnitus. Simply delivering information via a single medium (especially text) does not sustain attention nor positively influence patients with tinnitus. Thanks to the flexibility and expandability of smart pad applications, we can now mix graphic, audio, video, and text content to maximize treatment effect even in less motivated or elderly patients.

Interestingly, the smart-TRT group had a slightly better treatment outcome (3-month ΔTHI of –23.3 points) than the conv-TRT group and in former publications on conv-TRT. Other studies reported a ΔTHI outcome of conv-TRT of –8.3 or –14.5 points at 3 months [33,34]. This is very similar to our results in the conv-TRT group (ΔTHI of –16.8 points). The interpretation of why smart-TRT is slightly better can be controversial. The most probable explanation is that this difference is not statistically nor clinically significant. Another explanation can be that smart-TRT is better because the educational interventions were delivered by 3 different specialists. For conv-TRT, a single health provider was in charge and provided a continued series of directive counseling. This can ensure a good patient-doctor relationship, but the counseling technique and content can become monotonous after several visits. In contrast, 3 different specialists can provide different insights and opportunities for motivation, despite delivering the content though a smart pad. The basic idea and general approach in treating tinnitus were the same, but details on how to deliver the idea were different between specialists. Also, the patients might have felt more confident about their treatment program because 3 different specialists spoke with one voice and repeated the main idea every time.

### Limitations

There are several limitations in this study. First, this was not a randomized trial. Although this was a prospective study and the smart-TRT group was enrolled according to the predetermined plan, the conv-TRT group had pre-existing data that were closely matched to the smart-TRT group. However, we believe this point did not undermine the main idea of this study. This is because (1) all the baseline demographics were very similar between the 2 groups and (2) the patients were recruited from the same institute during a similar period, managed by the same medical personnel, and evaluated using identical questionnaires at identical time points. Second, the follow-up duration was not long enough. Since tinnitus is a chronic disorder, the treatment effect should be followed for several months to years. Unfortunately, we were only able to follow the patients up to 3 months. There is a possibility that the results could be different in the long term. However, according to our previous studies, the short-term effect of TRT can also provide clinically important information [1,35]. Third, both smart-TRT and conv-TRT were not able to decrease the perceived sound itself. That is, there was no treatment effect in the VAS category of tinnitus loudness. TRT may only be effective in controlling the distress caused by tinnitus. This is different from recently introduced treatments that can also control the loudness of tinnitus [17,36].

### Conclusions

TRT could be effectively delivered to patients with tinnitus using smart devices. TRT-based smart devices could save the time and cost associated with conventional in-person therapy. These methods have gained attention during the COVID-19 era for the potential to decrease the chance of viral spread.

## Abbreviations

BDI: Beck Depression Inventory
conv-TNT: conventional TRT
ICT: information and communication technology
KHIDI: Korea Health Industry Development Institute
NRF: National Research Foundation
PSQI: Pittsburgh Sleep Quality Index
smart-TNT: smart device–based TRT
SMG-SNU: Seoul Metropolitan Government-Seoul National University
STAI: State-Trait Anxiety Inventory
THI: Tinnitus Handicap Inventory
TRT: tinnitus retraining therapy
VAS: visual analogue scale
